# The Association between Dietary Iron Intake, SNP of the MTNR1B rs10830963, and Glucose Metabolism in Chinese Population

**DOI:** 10.3390/nu15081986

**Published:** 2023-04-20

**Authors:** Liping Shen, Zhengyuan Wang, Jiajie Zang, Hong Liu, Ye Lu, Xin He, Chunfeng Wu, Jin Su, Zhenni Zhu

**Affiliations:** 1 Division of Health Risk Factors Monitoring and Control, Shanghai Municipal Center for Disease Control and Prevention, 1380 West Zhongshan Road, Shanghai 200336, China; shenliping@scdc.sh.cn (L.S.);; 2 Division of Non-Communicable Diseases Prevention and Control, Shanghai Municipal Center for Disease Control and Prevention, 1380 West Zhongshan Road, Shanghai 200336, China; 3 Department of Profession Management, Shanghai Municipal Center for Disease Control and Prevention, 1380 West Zhongshan Road, Shanghai 200336, China

**Keywords:** dietary iron, MTNR1B, single-nucleotide polymorphism, rs10830963, glucose metabolism

## Abstract

Type 2 diabetes is associated with both dietary iron intake and single-nucleotide polymorphism (SNP) of intronic rs10830963 in melatonin receptor 1B (MTNR1B); however, it is unclear whether they interact. The aim of this study was to examine the associations between dietary iron intake, SNP of rs10830963, and glucose metabolism. Data were obtained from the Shanghai Diet and Health Survey (SDHS) during 2012–2018. Standardized questionnaires were carried out through face-to-face interviews. A 3-day 24 h dietary recall was used to evaluate dietary iron intake. Anthropometric and laboratory measurements were applied. Logistic regression and general line models were used to evaluate the association between dietary iron intake, SNP of the MTNR1B rs10830963, and glucose metabolism. In total, 2951 participants were included in this study. After adjusting for age, sex, region, years of education, physical activity level, intentional physical exercise, smoking status, alcohol use, and total energy, among G allele carriers, dietary iron intake was associated with a risk of elevated fasting glucose, higher fasting glucose, and higher HbA1c, while no significant results were observed among G allele non-carriers. The G allele of intronic rs10830963 in MTNR1B potentially exacerbated unfavorable glucose metabolism with the increasing dietary iron intake, and it was possibly a risk for glucose metabolism homeostasis in the Chinese population.

## 1. Introduction

Type 2 diabetes (T2D) is an ever-increasing issue worldwide, and its prevalence has reached alarming levels. Results from the International Diabetes Federation (IDF) show that nearly half a billion people (9.3% of adults 20–79 years) were living with diabetes worldwide in 2019; the highest number of diabetes cases was in China (116.4 million), and the number will increase to 147.2 million by 2045 [1]. The prevalence of diabetes in adults aged 20–79 years in China has increased from 9.7% in 2007 to 12.8% in 2017 [2,3].

T2D is a multifactorial disease resulting from dietary and behavioral risk factors as well as variations in gene mutations. Iron plays a direct and causal role in diabetes pathogenesis mediated both by beta-cell failure and insulin resistance [4]. High levels of dietary and body iron are associated with increased levels of oxidative stress that may elevate the risk of T2D [5,6,7]. Melatonin receptor 1B, encoded by MTNR1B, mediates melatonin regulating insulin secretion and glucose concentration [8]. Melatonin was known to inhibit insulin secretion [9]. SNP of the MTNR1B rs10830963 leads to the substitution of single nucleotides at specific positions, resulting in different transcribed peptides with various functions. It was found that participants carrying the minor G allele of rs10830963 in the MTNR1B were associated with higher fasting plasma glucose concentration and glycohemoglobin A1c (HbA1c), increased homeostasis model assessment of insulin resistance (HOMA2-IR), reduced beta-cell function, and increased risk of T2D [10,11,12,13,14].

Although T2D is well described in terms of its hallmarks of insulin resistance and beta-cell failure, the proximal causes of T2D, the mechanisms underlying its genetic predisposition, and dietary iron intakes in interaction with variations in multiple loci of genes remain largely unknown. Recent reports have demonstrated a relationship between T2D-associated MTNR1B rs10830963 variants and transcriptional enhancer activity, further influencing liver function and leading to abnormal metabolism [15]. Iron overload induces liver injury [16]. Whether the combination of the rs10830963 allele and iron increases the risk of abnormal glucose metabolism is unclear. The aim of this study was to examine the associations between dietary iron intake and glucose metabolism and its interaction with the SNP of MTNR1B rs10830963. Over 100 million people are living with diabetes in China; therefore, there is an urgent need to develop and implement strategies for the precise prevention and control of diabetes.

## 2. Materials and Methods

### 2.1. Study Participants

**Figure 1:**
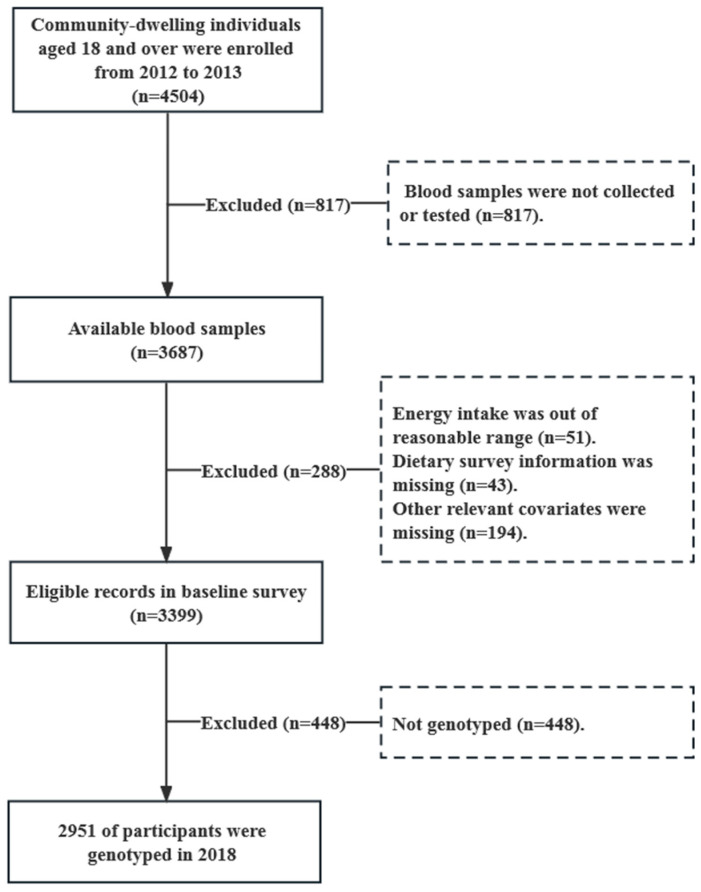
Flow chart of the study participants.

This study was based on the Shanghai Diet and Health Survey (SDHS), which was conducted from 2012 to 2013; the participants were followed up in 2019. It was a cross-sectional study, and the methodology of this study has been described previously [17]. Briefly, a multi-stage stratified cluster random sampling was used to select participants from 54 communities across Shanghai from 2012 to 2013 (Figure 1). Initially, 4504 community-dwelling individuals aged over 18 years old were enrolled. We excluded the participants with uncollected or untested blood samples (*n* = 817), unreasonable energy intakes (less than 300 kcal/d or more than 3500 kcal/d (*n* = 51), missing dietary survey information (*n* = 43) or other relevant covariates (*n* = 194), and non-genotyped participants (*n* = 448). Finally, 2951 participants genotyped in 2018 were included in this study; these were followed up in 2019 (Table S1).

This study was approved by the Ethics Review Board of the Shanghai Municipal Center for Disease Control and Prevention in 2012. Informed consent was obtained from all subjects involved in this study.

### 2.2. Dietary Assessment

Individual dietary intakes were obtained from 3 consecutive days using the 3-day 24 h recall method (2 weekdays and 1 weekend day) by trained investigators. In addition, food and condiment intakes from the home inventory, food purchased from markets or picked from gardens, and food waste were recorded and quantified during this 3-day survey period. Based on the sorted food names, ingredients, and exact weight of the 3-day 24 h diet record, dietary energy and iron intake were calculated using The Chinese Food Composition Table [18,19]. Dietary supplements were excluded from total dietary iron intake. RNI values for iron in adults were 12 mg/d in males, 20 mg/d in females aged 18–49 (excluding pregnant and lactating women), and 12 mg/d in females aged 50 and above recommended by Chinese Dietary Reference Intakes (DRIs) 2013 [20]. Dietary total iron intake was quadratically categorized as <12.82, 12.82–16.59, 16.59–22.07, and ≥22.07 mg/d.

### 2.3. Laboratory Measurements

Venous samples were collected by trained investigators at the local community health centers using uniform instruments after an overnight fast of 12 h. The collected blood samples were immediately centrifuged at 3000 r/min, aliquoted, and stored at −80 °C until analysis. The blood HbA1c concentrations were determined using a HITACHI 7080 Automatic Biochemical Analyzer with reagents obtained from Wako Pure Chemical Industries, Ltd. (Tokyo, Japan); fasting insulin concentrations were measured using a Chemiluminescence Immune Detection System (ACCESS 2, Beckman Coulter, Los Angeles, CA, USA). All these measurements were conducted in the laboratory of the Shanghai Municipal Center for Disease Control and Prevention from 2012 to 2013.

### 2.4. Genotyping

During the 2012–2013 fieldwork, white blood cells were stored at −80 °C after collection. DNA was first purified from the white blood cells in 2018 with the use of the magnetic bead method by the Universal Genomic DNA Extraction Kit (type DP705–02 from TIANGEN, Beijing, China). Genotyping was performed using a SNaPshot Multiplex System on a genetic inheritance analyzer (type 3730XL from Applied Biosystems, Waltham, MA, USA) for the SNP rs10830963. The electropherograms were analyzed using GeneMapper ID-X software (Thermo Fisher Scientific, Waltham, MA, USA).

### 2.5. Identification of Elevated Fasting Glucose and Calculation of HOMA2-IR

Elevated fasting glucose was defined as fasting glucose ≥100 mg/dL (about 5.56 mmol/L) or on glucose-lowering drug treatment [21]. We calculated the homeostasis model assessment of insulin resistance (HOMA2-IR) using the HOMA2 Calculator v2.2.3, which is available on the website of the Oxford Centre for Diabetes, Endocrinology and Metabolism (www.dtu.ox.ac.uk, accessed on 10 February 2022) [22].

### 2.6. Potential Confounders

Individuals’ characteristics and lifestyle information were collected by an interviewer-administered questionnaire, including participants’ age, gender, annual household income (computed by dividing the total family annual income by the number of family members), educational level (reported as years of education), dietary energy intake, physical activity level (sedentary, moderate, and vigorous), intentional physical exercise (defined as physical exercise performed to maintain health or fitness), smoking status (never, former, and current), and alcohol use (four categories, including lifetime abstainers who had not ever consumed any alcoholic beverages, nonheavy drinkers/social drinkers who consumed 5+ standard drinks once (1 day) in every 1-week period, infrequent heavy drinkers/binge drinkers who consumed 5+ standard drinks on 2–3 days in every 1–week period, and frequent heavy drinkers who consumed 5+ standard drinks on at least 4 days in every 1-week period).

### 2.7. Statistical Analysis

The odds ratios (ORs) and 95% confidence intervals (CIs) of elevated fasting glucose were analyzed using logistic regression models, where the occurrence of elevated fasting glucose was treated as the dependent variable, and a product term of at least one G allele presence of rs10830963 (a binary variable, coded as 1 for presence and 0 for non-presence) and the quartiles of dietary iron intake were the independent variables. The β and 95% CI were used to evaluate the association between the dependents of fasting glucose, HbA1c, and HOMA2-IR, and independents of dietary iron intake and the SNP rs10830963, by using general linear regression, after excluding 170 participants receiving glucose-lowering treatment. All the data were analyzed using SAS 9·4 release (SAS Institute Inc., Cary, NC, USA). Statistical tests were two-sided, and a *p*-value less than 0.05 was considered statistically significant.

## 3. Results

### 3.1. Characteristics of the Participants

The characteristics of the participants are shown in Table 1. In total, 2951 participants were included in this study (47.5% were males), and 2004 (67.9%) of them were G allele carriers.

### 3.2. Genotypes of the MTNR1B rs10830963

The distribution of rs10830963 genotypes among all participants was 17.7% for GG, 50.3% for GC, and 32.0% for CC (Table 2). The minor allele frequency (MAF) of the G allele was 42.9% (43.2% of males and 42.5% of females).

### 3.3. Associations between Dietary Iron and Risk on the Glucose Metabolism When Stratified by G Allele on the rs10830963 Site of MTNR1B Gene

#### 3.3.1. Associations between Dietary Iron and Risk on Elevated Fasting Glucose Stratified by the rs10830963 Risk Allele in the MTNR1B Gene

**Figure 2:**
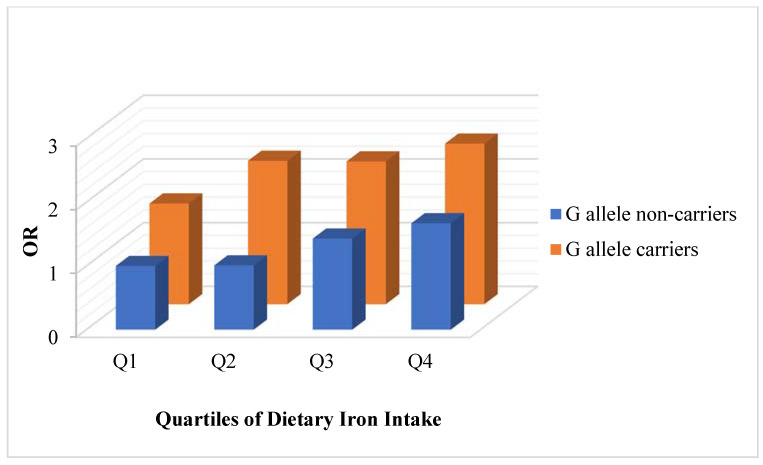
Associations between dietary iron and risk of elevated fasting glucose among all participants stratified by G allele on the rs10830963 site of the MTNR1B gene. Data were presented as ORs for elevated fasting glucose according to the quartiles of dietary iron intake. The subgroup of G allele non-carriers in the lowest quartile of dietary iron intake (<12.82 mg/day) was set to be the reference, meaning OR = 1.00. The other seven subgroups were compared with the reference subgroup.

After adjusting for age, sex, region, years of education, physical activity level, intentional physical exercise, smoking status, alcohol use, and total energy, a borderline significant trend was observed across the quartiles of total dietary iron and risk of elevated fasting glucose among G allele non-carriers (*p* = 0.066), while a linear trend was found among G allele carriers (*p* = 0.033) in all participants (Table 3 and Figure 2).

In all participants, compared with the subgroup of G allele non-carriers in the lowest quartile of dietary iron intake (<12.82 mg/day), the ORs (95% CI) for the elevated fasting glucose of G allele non-carriers were 1.01 (0.58, 1.75) in the second quartile (12.82–16.59 mg/day), 1.43 (0.82, 2.49) in the third quartile (16.59–22.07 mg/day), and 1.67 (0.87, 3.18) in the highest quartile (≥22.07 mg/day), whereas those of G allele carriers were 1.58 (1.01, 2.47) in the lowest quartile, 2.25 (1.63, 3.10) in the second quartile, 2.24 (1.59, 3.15) in the third quartile, and 2.52 (1.69, 3.76) in the highest quartile.

Compared with the subgroup of G allele non-carriers in the lowest quartile of dietary iron intake, the ORs (95% CI) for elevated fasting glucose of G allele carriers were 2.03 (0.93, 4.44), 2.67 (1.59, 4.47), 2.34 (1.39, 3.95), and 3.22 (1.82, 5.70) from the lowest to the highest quartile of dietary iron intake for males, whereas the ORs (95% CI) were 1.44 (0.82, 2.53), 2.12 (1.39, 3.23), 2.55 (1.61, 4.04), and 1.99 (1.10, 3.61) for females.

#### 3.3.2. Associations between Dietary Iron and Risk on Fasting Glucose Stratified by the rs10830963 Risk Allele in the MTNR1B Gene

In total, 170 participants who previously received glucose-lowering treatment in 2 weeks or in 12 months were excluded from the subsequent analysis. After adjusting for the same potential confounders, significant positive trends were observed across quartiles of total dietary iron and fasting glucose among G allele carriers in participants (*p* = 0.040) and females (*p* = 0.021), although no significant positive trends were observed in males (*p* = 0.442) (Table 4).

In all participants, compared with the subgroup of G allele non-carriers in the lowest quartile of dietary iron, the βs (95% CI) for fasting glucose of G allele carriers were 0.18 (0.01, 0.35) in the lowest quartile, 0.32 (0.16, 0.48) in the second quartile, 0.31 (0.14, 0.47) in the third quartile, and 0.40 (0.21, 0.60) in the highest quartile.

Compared with the subgroup of G allele non-carriers in the lowest quartile of dietary iron intake, the βs (95% CI) for the fasting glucose of G allele carriers were 0.24 (0.12, 0.60), 0.31 (0.03, 0.58), 0.29 (0.02, 0.56), and 0.37 (0.07, 0.67) from the lowest to the highest quartile of dietary iron intake for males, whereas the βs (95% CI) were 0.12 (0.05, 0.29), 0.32 (0.13, 0.51), 0.32 (0.10, 0.53), and 0.43 (0.17, 0.70) for females.

#### 3.3.3. Associations between Dietary Iron and Risk on HbA1c Stratified by the rs10830963 Risk Allele in the MTNR1B Gene

Among G allele carriers, after adjusting for the same confounders, a significant positive trend was observed across the quartiles of total dietary iron intake and HbA1c in all participants (*p* = 0.004) and in the female participants (*p* = 0.014) but not among the male participants (*p* = 0.101) (Table 5).

In all participants, compared with the subgroup of G allele non-carriers in the lowest quartile of dietary iron intake, the βs (95% CI) for fasting glucose of G allele carriers were 0.10 (−0.03, 0.22) in the lowest quartile, 0.30 (0.17, 0.43) in the second quartile, 0.33 (0.19, 0.47) in the third quartile, and 0.33 (0.17, 0.49) in the highest quartile.

Compared with the subgroup of G allele non-carriers in the lowest quartile of dietary iron intake, the βs (95% CI) for fasting glucose of G allele carriers were 0.19 (0.08, 0.46), 0.28 (0.04, 0.51), 0.34 (0.11, 0.57), and 0.39 (0.14, 0.65) from the lowest to the highest quartile of dietary iron intake for males, whereas the βs (95% CI) were 0.07 (0.07, 0.21), 0.16 (0.08, 0.39), 0.22 (0.01, 0.45) and 0.27 (0.02, 0.53) for females.

#### 3.3.4. Associations between Dietary Iron and Risk on HOMA2-IR Stratified by the rs10830963 Risk Allele in the MTNR1B Gene

After adjusting for the same confounders, no significant positive trend or significant difference was observed across the quartiles of total dietary iron intake and HOMA2-IR in all male and female participants (Table 6).

## 4. Discussion

We found that SNP rs10830963 of MTNR1B was associated with dietary iron intake and glucose metabolism. We assumed that the G allele of rs10830963 was a risk allele for glucose metabolism homeostasis. The G allele of intronic rs10830963 in MTNR1B potentially exacerbated unfavorable glucose metabolism with the increasing dietary iron intake. Few studies have reported these associations. Several gene-based association studies have shown that the G allele of intronic rs10830963 in MTNR1B conferred an increased risk of impaired fasting glycemia and T2D through an impaired glucose-stimulated insulin release [11,12]. Melatonin is strongly associated with glucose concentration and insulin release. The variant in MTNR1B could lead to functional and activity changes in melatonin, contributing to the etiopathogenesis of abnormal glucose metabolism. Research has demonstrated that the SNP of the MTNR1B rs10830963 increases FOXA2-bound enhancer activity in islet- and liver-derived cells and it is associated with the incidence of T2D [15]. FOXA2 is a pioneer factor that binds native chromatin and bookmarks genomic regions for transcriptional activity [23] and is involved in pancreatic and hepatic development [24,25]. The G allele of rs10830963 increases T2D risk through increased FOXA2-bound enhancer activity, potentially mediated through NEUROD1 binding in islets, and consequently, higher expression of MTNR1B [15]. Dietary iron accounted for diabetes. Studies have shown that high intake levels of dietary iron intake took part in diabetes pathogenesis mediated both by beta-cell failure and insulin resistance and elevated oxidative stress [4,7,26]. As an important metabolic organ of the human body, the liver is closely related to metabolism, and it orchestrates systemic iron balance by producing and secreting hepcidin [27]. Excess iron levels cause liver injury [16]. Therefore, we speculated that the G allele of rs10830963 and dietary iron intake might exacerbate unfavorable glucose metabolism via co-operatively influencing the liver metabolism pathway. Moreover, the synergistic mechanism warrants further study.

The MAF of the G allele in the MTNR1B SNP rs10830963 site is more dominant in Chinese populations than in Caucasian populations. The study found that the MAF of the G allele in the MTNR1B SNP rs10830963 was 42.3% in a Chinese population [11]. We found the proportion to be 42.8% in our study population, which was considerably higher than in Caucasian populations (26.7%) [28]. The 1000 Genomes project also demonstrated that the G allele frequency was 26.0% worldwide: 2.7% in Africa, 19.3% in America, 28.8% in Europe, and approximately 42.0% in Asia [29]. Diabetes is a serious, long-term condition with a major impact on the lives and well-being of individuals, families, and societies. There is the highest number of diabetes cases and considerably higher risk G allele frequency in Chinese populations [1,29]. It was of great significance to further study the influence of the MTNR1B rs10830963 SNP on glucose metabolism in Chinese populations to precisely prevent and control T2D.

There are some limitations to the current study. First of all, due to the cross-sectional nature of this study, we cannot conclude the causal inferences between MTNR1B SNP rs10830963 and glucose metabolism. Furthermore, individual dietary intakes of energy and iron were obtained using the 3-day 24 h recall method; thus, recall bias and information bias from self-reported dietary intakes may lead to biased estimates. Finally, the HOMA2 model is a structural computer model of the glucose–insulin feedback system in the homeostatic state and is used to measure insulin resistance. Insulin concentrations ranging from 20 to 400 pmol/L were acceptable steady-state peptide input values in the model. However, there were apparently healthy participants in the study population (insulin concentrations below 20 pmol/L, accounting for 24.8%). Therefore, the values might have been relatively unreliable when we used this model.

## 5. Conclusions

Polymorphisms of rs10830963 are more common in Chinese populations than in Caucasian populations. SNP rs10830963 and dietary iron intake were associated with glucose metabolism in the current study. The G allele of intronic rs10830963 in MTNR1B potentially exacerbated unfavorable glucose metabolism with increasing dietary iron intakes. We assumed that the G allele of rs10830963 was a possible risk for glucose metabolism homeostasis in the Chinese population.

## Figures and Tables

**Table 1:** Characteristics of the participants, stratified by G allele presence on the rs10830963 site of the gene MTNR1B.

	All			G allele Non-Carriers			G allele Carriers ^1^		
	Male	Female	All	Male	Female	All	Male	Female	All
*n* (%)	1403 (47.5)	1548 (52.5)	2951 (100.0)	452 (47.7)	495 (52.3)	947 (32.1)	951 (47.5)	1053 (52.5)	2004 (67.9)
Age, %									
15−44 years	426 (30.4)	503 (32.5)	929 (31.5)	125 (27.6)	169 (34.1)	294 (31.0)	301 (31.7)	334 (31.7)	635 (31.7)
45–59 years	502 (35.8)	555 (35.9)	1057 (35.8)	168 (37.2)	185 (37.4)	353 (37.3)	334 (35.1)	370 (35.2)	704 (35.1)
60− years	475 (33.8)	490 (31.6)	965 (32.7)	159 (35.2)	141 (28.5)	300 (31.7)	316 (33.2)	349 (33.1)	665 (33.2)
Annual Household Income, %									
Above average level (RMB > 60,000)	772 (55.0)	879 (56.8)	1651 (56.0)	229 (50.7)	287 (58.0)	516 (54.5)	543 (57.1)	592 (56.2)	1135 (56.6)
Average level (RMB 30,000–59,999)	453 (32.3)	466 (30.1)	919 (31.1)	164 (36.3)	142 (28.7)	306 (32.3)	289 (30.4)	324 (30.8)	613 (30.6)
Below average level (RMB < 30,000)	106 (7.6)	132 (8.5)	238 (8.0)	40 (8.8)	37 (7.4)	77 (8.1)	66 (6.9)	95 (9.0)	161 (8.0)
No answer	72 (5.1)	71 (4.6)	143 (4.9)	19 (4.2)	29 (5.9)	48 (5.1)	53 (5.6)	42 (4.0)	95 (4.7)
Years of Education, years (SD)	10.3 (4.0)	9.1 (4.8)	9.7 (4.5)	10.2 (4.0)	9.2 (4.7)	9.7 (4.4)	10.3 (4.0)	9.1 (4.9)	9.7 (4.5)
Physical Activity Level, %									
Sedentary	1119 (79.8)	1383 (89.4)	2502 (84.8)	368 (81.4)	439 (88.7)	807 (85.2)	751 (79.0)	944 (89.7)	1695 (84.6)
Moderate	244 (17.4)	151 (9.8)	395 (13.4)	70 (15.5)	52 (10.5)	122 (12.9)	174 (18.3)	99 (9.4)	273 (13.6)
Vigorous	40 (2.8)	13 (0.8)	53 (1.8)	14 (3.1)	4 (0.8)	18 (1.9)	26 (2.7)	9 (0.9)	35 (1.8)
Intentional Physical Exercise, %	353 (25.3)	388 (25.1)	741 (25.2)	127 (28.0)	127 (25.7)	254 (26.8)	226 (23.9)	261 (24.8)	487 (24.4)
Smoking Status, %									
Never smoked	571 (40.7)	1527 (98.6)	2098 (71.1)	185 (41.0)	487 (98.4)	672 (71.0)	386 (40.6)	1040 (98.7)	1426 (71.2)
Former smoker	147 (10.5)	6 (0.4)	153 (5.2)	61 (13.5)	3 (0.6)	64 (6.8)	86 (9.1)	3 (0.3)	89 (4.4)
Current smoker	684 (48.8)	15 (1.0)	699 (23.7)	206 (45.5)	5 (1.0)	211 (22.2)	478 (50.3)	10 (1.0)	488 (24.4)
Alcohol use, %									
Lifetime abstainers	828 (64.2)	1408 (94.9)	2236 (80.6)	274 (66.8)	449 (95.6)	723 (82.1)	554 (63.1)	959 (94.6)	1513 (80.0)
Nonheavy drinkers	353 (27.4)	68 (4.5)	421 (15.2)	111 (26.9)	18 (3.8)	129 (14.6)	242 (27.6)	50 (4.9)	292 (15.4)
Infrequent heavy drinkers	29 (2.3)	4 (0.3)	33 (1.2)	5 (1.2)	2 (0.4)	7 (0.8)	24 (2.7)	2 (0.2)	26 (1.4)
Frequent heavy drinkers	79 (6.1)	4 (0.3)	83 (3.0)	21 (5.1)	1 (0.2)	22 (2.5)	58 (6.6)	3 (0.3)	61 (3.2)
Dietary Intake									
Energy, kcal/day (SD)	1945.8 (918.4)	1608.9 (776.4)	1769.1 (863.3)	1882.5 (746.8)	1580.9 (649.4)	1724.9 (713.3)	1975.8 (988.6)	1622.1 (829.4)	1790.1 (925.2)
Total iron, mg/day (SD)	22.5 (21.5)	17.8 (11.4)	20.0 (17.1)	21.9 (19.2)	17.8 (10.2)	19.8 (15.3)	22.7 (22.5)	17.8 (11.9)	20.0 (17.9)
Glucose Metabolism Index									
Elevated fasting glucose, %	351 (25.0)	326 (21.1)	677 (22.9)	93 (20.5)	96 (19.4)	189 (19.9)	258 (27.0)	230 (21.8)	488 (24.3)
Fasting Glucose, mmol/L	5.2 (1.2)	5.1 (1.1)	5.2 (1.1)	5.1 (1.1)	5.1 (1.0)	5.1 (1.1)	5.2 (1.1)	5.2 (1.1)	5.2 (1.1)
HbA1c, %	5.7 (1.0)	5.7 (0.9)	5.7 (1.0)	5.6 (1.0)	5.6 (0.8)	5.6 (0.9)	5.8 (1.0)	5.8 (0.9)	5.8 (1.0)
HOMA2-IR	0.6 (0.5)	0.7 (0.6)	0.7 (0.6)	0.6 (0.5)	0.8 (0.7)	0.7 (0.6)	0.7 (0.5)	0.7 (0.6)	0.7 (0.5)

Abbreviations: renminbi, RMB; standard deviation, SD; glycated hemoglobin A1c, HbA1c; homeostasis model assessment of insulin resistance, HOMA2-IR. ^1^ Those who at least have one G allele on the rs1080963 site of the gene MTNR1B, including GG and GC.

**Table 2:** Genotypes of the MTNR1B rs10830963 in the study participants.

	Frequency (%)		
	All (*n* = 2951)	Male (*n* = 1403)	Female (*n* = 1548)
Genotype			
GG	17.6	18.4	16.9
GC	50.3	49.4	51.1
CC	32.1	32.2	32.0
MAF			
G	42.8	43.1	42.5

Abbreviations: minor allele frequency, MAF.

**Table 3:** ORs (95% CIs) for elevated fasting glucose according to the quartiles of dietary iron stratified by the rs10830963 risk allele in the MTNR1B gene among the participants ^1^.

			Quartiles of Dietary Iron Intake (mg/day), ORs (95% CI) ^2^				
			Q1	Q2	Q3	Q4	*p*-Value for Trend ^3^
Total Iron Intake (mg/day)			<12.82	12.82–16.59	16.59–22.07	≥22.07	
*n*			736	739	740	736	
Elevated fasting glucose							
All	Model 1	G allele non-carriers	Reference	1.03 (0.62, 1.70)	1.53 (0.95, 2.45)	1.76 (1.10, 2.82)	0.005
		G allele carriers	1.44 (0.95, 2.18)	1.88 (1.39, 2.54)	1.75 (1.29, 2.38)	1.71 (1.26, 2.32)	0.375
	Model 2	G allele non-carriers	Reference	1.01 (0.58, 1.75)	1.43 (0.82, 2.49)	1.67 (0.87, 3.18)	0.066
		G allele carriers	1.58 (1.01, 2.47)	2.25 (1.63, 3.10)	2.24 (1.59, 3.15)	2.52 (1.69, 3.76)	0·033
Male	Model 1	G allele non-carriers	Reference	0.78 (0.35, 1.72)	1.46 (0.71, 3.00)	1.61 (0.79, 3.25)	0·046
		G allele carriers	1.75 (0.89, 3.46)	2.01 (1.25, 3.23)	1.68 (1.07, 2.65)	2.04 (1.32, 3.16)	0.667
	Model 2	G allele non-carriers	Reference	0.91 (0.37, 2.23)	1.35 (0.55, 3.30)	2.48 (0.90, 6.85)	0.034
		G allele carriers	2.03 (0.93, 4.44)	2.67 (1.59, 4.47)	2.34 (1.39, 3.95)	3.22 (1.82, 5.70)	0.180
Female	Model 1	G allele non-carriers	Reference	1.27 (0.66, 2.43)	1.53 (0.81, 2.88)	1.84 (0.97, 3.51)	0.051
		G allele carriers	1.27 (0.75, 2.14)	1.80 (1.22, 2.67)	1.96 (1.30, 2.95)	1.37 (0.88, 2.15)	0.410
	Model 2	G allele non-carriers	Reference	1.12 (0.53, 2.37)	1.53 (0.72, 3.25)	1.19 (0.48, 2.97)	0.512
		G allele carriers	1.44 (0.82, 2.53)	2.12 (1.39, 3.23)	2.55 (1.61, 4.04)	1.99 (1.10, 3.61)	0.095

^1^ G allele presence on rs10830963 was coded as 1 for presence and 0 for non-presence. Model 1 was adjusted for age and sex. Model 2 was adjusted for age, sex, region, years of education, physical activity level, intentional physical exercise, smoking status, alcohol use, and dietary total energy intake. ^2^ OR (95% CI) represents the risk of elevated fasting glucose occurrence of the current dietary iron intake range compared with the reference group. ^3^ The *p*-value for the trend was examined using the medians in each quartile of dietary iron intake.

**Table 4:** βs (95% CI) for fasting glucose according to the quartiles of dietary iron, stratified by the G allele of rs10830963 in the MTNR1B gene among the participants ^1^.

			Quartiles of Dietary Iron Intake (mg/day), βs (95% CI) ^2^				
			Q1	Q2	Q3	Q4	*p*-Value for Trend ^3^
Total Iron Intake (mg/day)			<12.82	12.82–16.59	16.59–22.07	≥22.07	
*n* ^4^			691	707	691	692	
Fasting glucose							
All	Model 1	G allele non-carriers	Reference	0.08 (−0.12, 0.29)	0.13 (−0.07, 0.34)	0.28 (0.08, 0.49)	0.007
		G allele carriers	0.16 (0.00, 0.33)	0.25 (0.11, 0.40)	0.24 (0.09, 0.38)	0.27 (0.12, 0.42)	0.185
	Model 2	G allele non-carriers	Reference	0.02 (−0.20, 0.25)	0.02 (−0.21, 0.26)	0.17 (−0.11, 0.45)	0.288
		G allele carriers	0.18 (0.01, 0.35)	0.32 (0.16, 0.48)	0.31 (0.14, 0.47)	0.40 (0.21, 0.60)	0.040
Male	Model 1	G allele non-carriers	Reference	−0.08 (−0.44, 0.27)	0.07 (−0.27, 0.41)	0.26 (−0.07, 0.60)	0.043
		G allele carriers	0.20 (−0.13, 0.52)	0.20 (−0.05, 0.45)	0.19 (−0.05, 0.43)	0.24 (0.00, 0.48)	0.708
	Model 2	G allele non-carriers	Reference	−0.07 (−0.46, 0.31)	−0.01 (−0.42, 0.39)	0.34 (−0.13, 0.81)	0.121
		G allele carriers	0.24 (−0.12, 0.60)	0.31 (0.03, 0.58)	0.29 (0.02, 0.56)	0.37 (0.07, 0.67)	0.442
Female	Model 1	G allele non-carriers	Reference	0.18 (−0.07, 0.42)	0.16 (−0.09, 0.40)	0.25 (−0.01, 0.50)	0.070
		G allele carriers	0.15 (−0.03, 0.33)	0.30 (0.11, 0.46)	0.27 (0.08, 0.47)	0.30 (0.10, 0.50)	0.138
	Model 2	G allele non-carriers	Reference	0.07 (−0.20, 0.33)	0.01 (−0.28, 0.30)	−0.01 (−0.36, 0.34)	0.908
		G allele carriers	0.12 (−0.05, 0.29)	0.32 (0.13, 0.51)	0.32 (0.10, 0.53)	0.43 (0.17, 0.70)	0.021

^1^ Model 1 was adjusted for age and sex. Model 2 was adjusted for age, sex, region, years of education, physical activity level, intentional physical exercise, smoking status, alcohol use, and dietary total energy intake. ^2^ β represents the partial correlation coefficients in the models, which means an elevated value of fasting glucose (mmol/L) for the subgroup of the specific dietary iron intake and the allele presence on the rs10830963 compared with the reference group. ^3^ The *p*-value for the trend was examined using the medians in each quartile of dietary iron intake. ^4^ Participants previously receiving glucose-lowering treatment (*n* = 170) were excluded from the analysis.

**Table 5:** βs (95% CI) for HbA1c according to the quartiles of dietary iron stratified by the rs10830963 risk allele in the MTNR1B gene among the participants ^1^.

			Quartiles of Dietary Iron Intake (mg/day), βs (95% CI) ^2^				
			Q1	Q2	Q3	Q4	*p*-Value for Trend ^3^
Total Iron Intake (mg/day)			<12.82	12.82–16.59	16.59–22.07	≥22.07	
*n* ^4^			691	707	691	692	
HbA1c							
All	Model 1	G allele non-carriers	Reference	0.09 (−0.09, 0.27)	0.04 (−0.13, 0.22)	0.13 (−0.05, 0.30)	0.259
		G allele carriers	0.07 (−0.06, 0.20)	0.24 (0.12, 0.37)	0.27 (0.14, 0.39)	0.22 (0.10, 0.34)	0.019
	Model 2	G allele non-carriers	Reference	0.06 (−0.13, 0.24)	−0.05 (−0.24, 0.15)	0.03 (−0.21, 0.26)	0.890
		G allele carriers	0.10 (−0.03, 0.23)	0.31 (0.18, 0.44)	0.34 (0.20, 0.47)	0.34 (0.17, 0.50)	0.003
Male	Model 1	G allele non-carriers	Reference	0.09 (−0.21, 0.40)	0.06 (−0.24, 0.37)	0.07 (−0.23, 0.36)	0.802
		G allele carriers	0.13 (−0.13, 0.39)	0.18 (−0.03, 0.40)	0.23 (0.02, 0.44)	0.22 (0.02, 0.43)	0.336
	Model 2	G allele non-carriers	Reference	0.09 (−0.25, 0.40)	−0.10 (−0.44, 0.24)	0.00 (−0.40, 0.40)	0.693
		G allele carriers	0.19 (−0.08, 0.46)	0.28 (0.04, 0.51)	0.34 (0.11, 0.57)	0.39 (0.14, 0.65)	0.101
Female	Model 1	G allele non-carriers	Reference	0.07 (−0.14, 0.27)	0.01 (−0.19, 0.22)	0.19 (−0.02, 0.41)	0.157
		G allele carriers	0.04 (−0.10, 0.18)	0.28 (0.14, 0.43)	0.30 (0.14, 0.46)	0.20 (0.04, 0.36)	0.018
	Model 2	G allele non-carriers	Reference	0.05 (−0.17, 0.27)	0.00 (−0.24, 0.24)	0.07 (−0.22, 0.36)	0.764
		G allele carriers	0.07 (−0.07, 0.21)	0.16 (−0.08, 0.39)	0.22 (−0.01, 0.45)	0.27 (0.02, 0.53)	0.014

^1^ Model 1 was adjusted for age and sex. Model 2 was adjusted for age, sex, region, years of education, physical activity level, intentional physical exercise, smoking status, alcohol use, and dietary total energy intake. ^2^ β represents the partial correlation coefficients in the models, which means the elevated value of HbA1c (%) for the subgroup of the specific dietary iron intake and the allele presence on the rs10830963 compared with the reference group. ^3^ The *p*-value for the trend was examined using the medians in each quartile of dietary iron intake. ^4^ Participants previously receiving glucose-lowering treatment (*n* = 170) were excluded from the analyses.

**Table 6:** βs (95% CI) for HOMA2-IR according to the quartiles of dietary iron stratified by the rs10830963 risk allele in the MTNR1B gene among the participants ^1^.

			Quartiles of Dietary Iron Intake (mg/day), βs (95% CI) ^2^				
			Q1	Q2	Q3	Q4	*p*-Value for Trend ^3^
Total Iron Intake (mg/day)			<12.82	(12.82–16.59)	(16.59–22.07)	≥22.07	
*n* ^4^			659	675	676	670	
HOMA2-IR							
All	Model 1	G allele non-carriers	Reference	−0.08 (−0.20, 0.04)	−0.03 (−0.15, 0.09)	−0.10 (−0.22, 0.02)	0.208
		G allele carriers	−0.09 (−0.19, 0.01)	−0.06 (−0.13, 0.01)	−0.04 (−0.11, 0.03)	−0.02 (−0.10, 0.05)	0.057
	Model 2	G allele non-carriers	Reference	−0.12 (−0.25, 0.01)	−0.09 (−0.23, 0.04)	−0.17 (−0.33, 0.00)	0.090
		G allele carriers	−0.12 (−0.22, −0.01)	−0.10 (−0.17, −0.02)	−0.08 (−0.16, 0.00)	−0.07 (−0.17, 0.02)	0.268
Male	Model 1	G allele non-carriers	Reference	−0.14 (−0.28, 0.01)	−0.04 (−0.18, 0.10)	−0.08 (−0.22, 0.06)	0.655
		G allele carriers	−0.08 (−0.23, 0.06)	−0.07 (−0.18, 0.04)	−0.03 (−0.14, 0.07)	−0.02 (−0.12, 0.08)	0.168
	Model 2	G allele non-carriers	Reference	−0.17 (−0.32, −0.01)	−0.07 (−0.24, 0.09)	−0.10 (−0.30, 0.09)	0.656
		G allele carriers	−0.12 (−0.28, 0.03)	−0.09 (−0.20, 0.03)	−0.07 (−0.19, 0.04)	−0.06 (−0.19, 0.06)	0.404
Female	Model 1	G allele non-carriers	Reference	−0.04 (−0.22, 0.15)	−0.03 (−0.21, 0.15)	−0.13 (−0.32, 0.06)	0.233
		G allele carriers	−0.10 (−0.23, 0.03)	−0.06 (−0.16, 0.03)	−0.05 (−0.15, 0.05)	−0.04 (−0.14, 0.07)	0.192
	Model 2	G allele non-carriers	Reference	−0.10 (−0.30, 0.10)	−0.12 (−0.34, 0.10)	−0.23 (−0.49, 0.04)	0.105
		G allele carriers	−0.13 (−0.27, 0.01)	−0.12 (−0.22, −0.02)	−0.09 (−0.20, 0.02)	−0.09 (−0.22, 0.05)	0.434

^1^ Model 1 was adjusted for age and sex. Model 2 was adjusted for age, sex, region, years of education, physical activity level, intentional physical exercise, smoking status, alcohol use, and dietary total energy intake. ^2^ β represents the partial correlation coefficients in the models, which means an elevated value of HOMA2-IR for the subgroup of the specific dietary iron intake and the allele presence on the rs10830963 compared with the reference group. ^3^ The *p*-value for the trend was examined using the medians in each quartile of dietary iron intake. ^4^ Participants previously receiving glucose-lowering treatment (*n* = 170) were excluded from the analyses. Additionally, 111 participants with unreasonable insulin levels (less than 5 pmol/L or more than 400 pmol/L) were excluded.

## Data Availability

The data presented in this study are available on reasonable request from the corresponding author. The data are not publicly available due to policy.

## Supplementary Materials

The following supporting information can be downloaded at: https://www.mdpi.com/article/10.3390/nu15081986/s1, Table S1: RRs (95% CI) for incident T2D was stratified by risk G allele of rs10830963 in the MTNR1B gene [30,31,32].

## Abbreviations

T2D, type 2 diabetes; SNP, single-nucleotide polymorphism; MTNR1B, melatonin receptor 1B; SDHS, Shanghai Diet and Health Survey; HbA1c, glycohemoglobin A1c; HOMA2-IR, homeostasis model assessment of insulin resistance; RMB, renminbi; SD, standard deviation; MAF, minor allele frequency; ORs, odds ratios; CIs, confidence intervals.
