# Supplementary material for: The Association between Dietary Iron Intake, SNP of the MTNR1B rs10830963, and Glucose Metabolism in Chinese Population
Source: Nutrients. 2023 Apr 20;15(8):1986. doi: 10.3390/nu15081986 (PMC10142655; doi:10.3390/nu15081986)
Supplement: Supplementary file 1 [file nutrients-15-01986-s001.zip › nutrients-2316818-supplementary.pdf]

Supplementary Materials

Results of self-reported diabetes analysis using data in the follow-up of 2019

A total of 2951 participants genotyped in 2018 and they were followed up in 2019. T2DM was self-reported by the participants in the follow-up. The participants were asked to report whether diagnosed T2DM once by the second-class or above hospital (Hospital in China is classified by its capacity. Second-class hospital means it provides service across several communities or in a district level.) and received any glucose-lowering drug treatment. And the relative risk (RR) and 95% CI were analyzed using logistic regression models to evaluate the association between incident T2DM with dietary iron intake and the SNP rs10830963, after excluding 166 participants who were previously diagnosed with T2DM using follow-up data collected in 2019. The self-reported T2DM incidence was 3.8% overall (3.3% among G allele non-carriers and 4.0% among G allele carriers) in 2019.

Association between SNP rs10830963 and risk of incident T2DM in the follow-up of 2019

A total of 166 participants who previously diagnosed with T2DM were excluded from the following analysis. After adjusting for age, sex, region, years of education, physical activity level, intentional physical exercise, smoking status, alcohol use and dietary total energy, no statistically significant result was observed across the quartiles of total dietary iron and risk of incident T2DM (**Table S1**).

Table S1. RRs (95% CI) for incident T2DM was stratified by risk G allele of rs10830963 in the MTNR1B gene[1]

			Quartiles of Dietary Iron Intake, RRs (95% CI) [2]				
			**Q1**	**Q2**	**Q3**	**Q4**	***p* Value for Trend**[3]
**Total Iron Intake (mg/day)**			<12.82	12.82-16·59	16.59-22.07	≥22.07	
**N**[4]			618	651	664	626	
**Incident T2DM**							
All	Model 1	G allele non-carriers	Reference	0.60(0.16,2.18)	1.64(0.59,4.52)	0.68(0.20,2.31)	0.969
		G allele carriers	1.11(0.42,2.94)	1.45(0.72,2.92)	1.24(0.60,2.53)	1.06(0.50,2.25)	0.771
	Model 2	G allele non-carriers	Reference	0.48(0.12,2.01)	1.57(0.44,5.59)	0.64(0.11,3.70)	0.799
		G allele carriers	0.97(0.36,2.63)	1.47(0.70,3.12)	1.31(0.58,2.97)	1.14(0.42,3.09)	0.805
Male	Model 1	G allele non-carriers	Reference	1.99(0.20,19.70)	4.77(0.58,39.19)	2.01(0.22,18.49)	0.500
		G allele	3.53(0.42,29.38)	3.95(1.48,10.55)	3.75(1.45,9.69)	2.51(0.93,6.79)	0.419

		carriers					
	Model 2	G allele non-carriers	Reference	1.13(0.09,13.50)	2.59(0.24,28.07)	0.85(0.05,14.62)	0.891
		G allele carriers	3.32(0.37,29.64)	4.38(1.50,12.87)	3.84(1.29,11.43)	2.43(0.65,9.00)	0.588
Female	Model 1	G allele non-carriers	Reference	0.25(0.03,2.19)	0.95(0.25,3.63)	0.32(0.04,2.78)	0.473
		G allele carriers	0.66(0.20,2.13)	0.99(0.36,2.70)	0.70(0.23,2.17)	1.00(0.33,3.02)	0.612
	Model 2	G allele non-carriers	Reference	0.31(0.03,3.19)	2.41(0.41,14.30)	2.01(0.13,32.25)	0.393
		G allele carriers	0.69(0.20,2.36)	1.31(0.45,3.88)	1.02(0.28,3.68)	1.88(0.40,8.82)	0.299

[1] G allele presence on rs10830963 was coded as 1 for presence and 0 for non-presence. Model 1 was adjusted for age and sex. Model 2 was adjusted for age, sex, region, years of education, physical activity level, intentional physical exercise, smoking status, alcohol use and dietary total energy intake.

[2] RR (95%CI) represents the risk of incident T2DM of each additional quartile of dietary iron intake reference to Q1

[3] The *p*-value for the trend was examined using the medians in each quartile of dietary iron intake.

[4] Participants previously diagnosed with T2DM (n =166) were excluded from the analysis. Information on diabetes was missing in 226 participants.

We followed up participants in 2019 and found that the self-reported incidence of T2DM was 3·8%, but no significant association was detected between the SNP rs10830963 in the MTNR1B and risk of incident T2DM (RR (95% CI): 1.27(0.79,2.05)). However, we assumed that the insignificant association may originate from relatively high undiagnosed proportion of T2DM in our study. Results from the International Diabetes Federation Diabetes 2019 shows that of the 463 million people living with diabetes, half (50.1%) are unaware of their condition. Higher proportions of undiagnosed diabetes were found in low- and middle-income countries, accounting for 84.3% of all undiagnosed people with diabetes worldwide [1]. Some studies also showed that carriers of the rs10830963 risk G allele had a higher risk of T2DM [2-4]. Therefore, there might be a high probability of significant association between carriers of the common intronic MTNR1B rs10830963 risk G with increased incident T2DM risk, if the incidence of newly diagnosed T2DM were not underestimated.

Reference

1. Saeedi P, Petersohn I, Salpea P, et al. Global and regional diabetes prevalence estimates for 2019 and projections for 2030 and 2045: Results from the International Diabetes Federation Diabetes Atlas, 9(th) edition. Diabetes Res Clin Pract. 2019;157:107843.
2. Tan X, Ciuculete DM, Schiöth HB, Benedict C. Associations between chronotype, MTNR1B genotype and risk of type 2 diabetes in UK Biobank. J Intern Med. 2020;287(2):189–96.
3. Jabbari F, Mohseni Bandpei A, Daneshpour MS, et al. Role of Air Pollution and rs10830963 Polymorphism on the Incidence of Type 2 Diabetes: Tehran Cardiometabolic Genetic Study. J Diabetes Res. 2020;2020:2928618.
4. Olsson L, Pettersen E, Ahlbom A, Carlsson S, Midthjell K, Grill V. No effect by the common gene variant rs10830963 of the melatonin receptor 1B on the association between sleep disturbances and type 2 diabetes: results from the Nord-Trøndelag Health Study. Diabetologia. 2011;54(6):1375–8.
